# Hashimoto's encephalopathy: A rare cause of refractory status epilepticus

**DOI:** 10.1111/cns.13562

**Published:** 2020-12-21

**Authors:** Zhenwei Guo, Xiaobing He, Guanghui Zhang, Chunyang Zhang, Aihua Tao, Bei Wang, Xinyi Wang, Luming Li, Mingli He

**Affiliations:** ^1^ Department of Neurology Lianyungang Clinical Medical College of Nanjing Medical University, the First People's Hospital of Lianyungang City Lianyungang China; ^2^ Department of Neurological Function Lianyungang Clinical Medical College of Nanjing Medical University, the First People's Hospital of Lianyungang City Lianyungang China

**Keywords:** anti‐TPO, Hashimoto's encephalopathy, SREAT, steroid‐responsive encephalopathy associated with autoimmune thyroiditis

## 1 INTRODUCTION

Hashimoto's encephalopathy (HE), also known as steroid‐responsive encephalopathy associated with autoimmune thyroiditis (SREAT), is a rare neurological disease that is poorly understood and difficult to diagnose.^1^ HE may present as an acute, subacute, or even chronic illness that is more common in women than men.^1^ The condition has been reported in pediatric, adult, and elderly populations throughout the world.^1^ The course of the disease is typically remitting and relapsing and can include cognitive and memory dysfunction, focal and generalized epileptic seizures, confusion, psychiatric disturbances, stroke‐like episodes, myoclonus, ataxia, tremor, and chorea form movements.^1^

Hashimoto's encephalopathy is generally considered to be an autoimmune encephalopathy; however, the exact pathophysiology of HE is unknown.^1^ HE is defined by the occurrence of anti‐thyroid antibodies (anti‐thyroid peroxidase antibody [anti‐TPO] and anti‐thyroglobulin antibody [anti‐TG]) and a positive response to steroids.^1^ While high titers of anti‐thyroid antibodies lead to a diagnosis of HE after other causes of encephalopathy have been excluded, it is not clear if the antibodies are involved in the clinical manifestations of the disease, and some authors suggest that a direct causal relationship between the thyroid antibodies and HE is unlikely.^1^, ^2^

Kothbauer‐Margreiter et al^3^ proposed 2 types of HE: a vasculitic type with stroke‐like episodes and a diffuse, progressive type. Seizures occur in both types, but more frequently in the diffuse type. Overall, 56% to 80% of patients with HE have seizures,^1^, ^4^ and 12% experience status epilepticus.^1^, ^5^ The 30‐day mortality rate of status epilepticus from any cause ranges from 7.6% to 22%, with the highest rates in the elderly.^6^

Herein, we present the case of a 14‐year‐old boy with HE who presented with refractory status epilepticus. The patient responded well to steroids, but required a combination of 4 anti‐seizure drugs to control his seizure activity.

## 2 CASE PRESENTATION

A 14‐year‐old boy was admitted to our emergency department (ED) in status epilepticus. Four weeks previously, he was seen at a neurology department because of generalized convulsive status epilepticus (GCSE), myoclonus, and hallucinations. The seizure frequency was about once 10 days, and the onset duration varied from 20 minutes to 60 minutes. He was hospitalized, but no seizures occurred during the hospitalization. Magnetic resonance imaging (MRI) of the brain revealed no obvious abnormalities. Electroencephalogram (EEG) monitoring for 24 hours indicated no epilepsy discharge waves. He was discharged on sodium valproate for seizure control. Twenty‐four hours before admission to our ED, he was again seen at the other hospital for GCSE. He had experienced 4 episodes, with an onset duration that varied from 10 minutes to 20 minutes. Sodium valproate, carbamazepine, and diazepam were administrated; however, the status epilepticus was not suspended. He was thus transferred to our ED. This study was approved by the institutional review board (IRB) of the First People's Hospital of Lianyungang City. Written informed consent was obtained from the patient. Written informed consent was obtained from the patient's father for the publication of any potentially identifiable images or data included in this article.

On examination in our ED, he was alert and his general examination was without abnormalities. Neurological evaluation only showed a bilateral Babinski sign, and he appeared to have normal cognitive development. He appeared to be of normal weight and height for his age, and his nutritional status appeared sufficient. He had no history of recent infections or flu‐like symptoms, injury, toxicosis, or academic achievement decline. He had a history of syncope several years prior, but the details of the event could not be obtained.

Routine laboratory tests, including a complete blood count (CBC), erythrocyte sedimentation rate, platelet count, tests of kidney (creatinine, urea nitrogen) and liver function (alanine aminotransferase, aspartate aminotransferase, albumin, direct bilirubin, indirect bilirubin, total bilirubin), and protein, lactate, and ammonia levels were normal. Urine toxicology was unremarkable. However, his glucose was slightly below the lower limit of normal (3.76 mmol/L, reference range: 3.9‐6.1 mmol/L). Testing for specific antibodies associated with autoimmune encephalitis including N‐methyl‐D‐aspartate (NMDA) receptor antibody, voltage‐gated potassium channel (VGKC) antibody, anti‐leucine‐rich glioma‐inactivated (LGI1) antibody, anti‐contactin‐associated protein‐like 2 (Caspr2) antibody, anti‐Hu antibody, and anti‐CRMP5/CV2 antibody was negative.

Thyroid function tests revealed a low thyroid‐stimulating hormone (TSH) level (0.06 mIU/L, reference range: 0.34‐5.60 mIU/L), but normal free triiodothyronine (FT3, 4.5 pmol/L, normal range: 3.8‐6.47) and free thyroxin (FT4, 1.36 pmol / L, normal range: 7.9‐17) levels. However, serum anti‐thyroid antibody levels were elevated. His anti‐TPO antibody level was 76.7 mIU/L (reference range: 0.25‐34 mIU/L), and his anti‐TG antibody level was 237.1 mIU/L (reference range: 0‐4 mIU/L).

**FIGURE 1:**
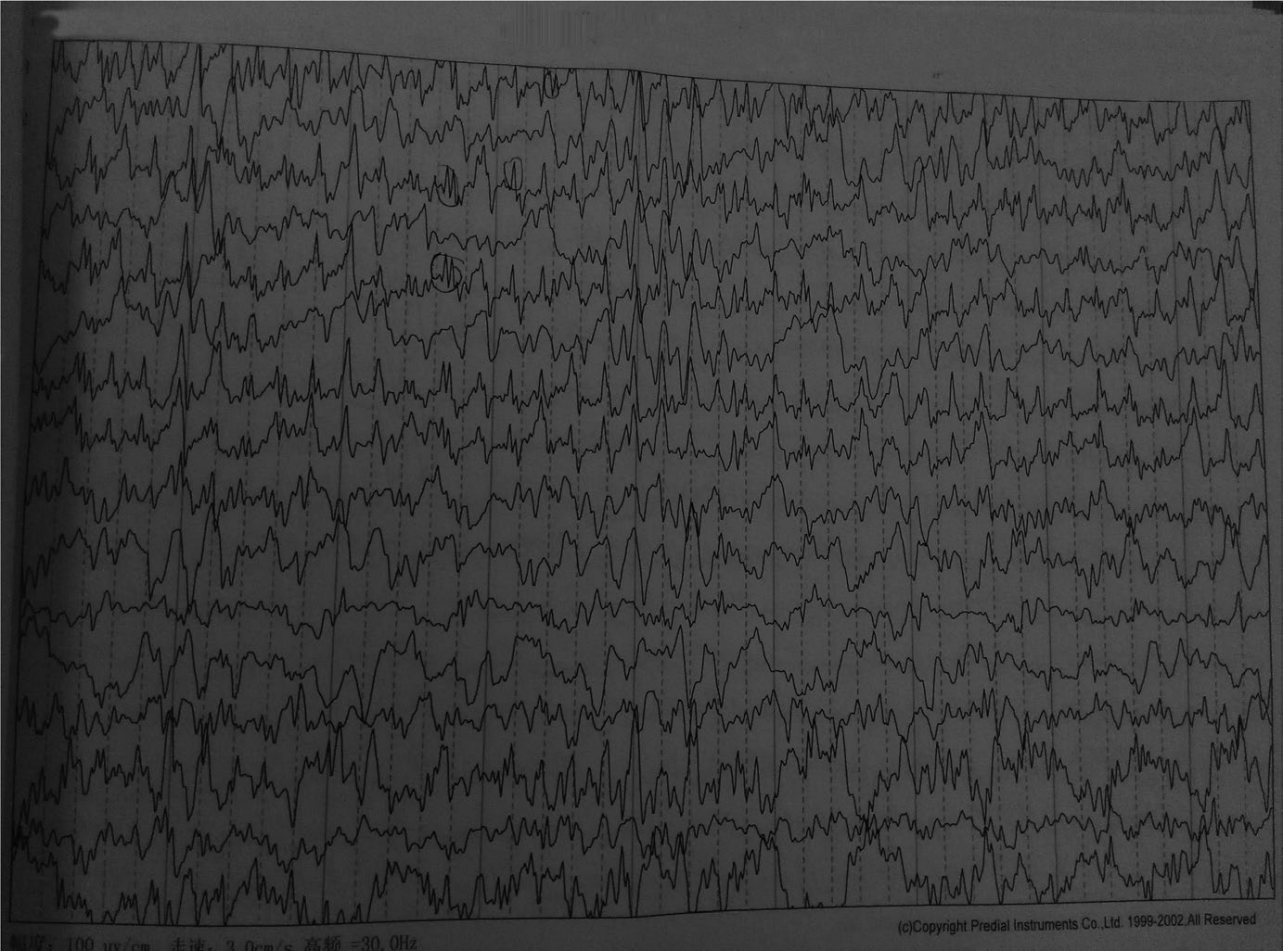
Electroencephalogram recorded 7 days after status epileptics showed many sharp waves over the left cerebral hemisphere

Cerebral computed tomography (CT) showed no signs of increased intracranial pressure, hemorrhage, or a space‐occupying lesion. MRI, including T1, T2, and diffusion‐weighted imaging (DWI) with apparent diffusion coefficient (ADC) mapping, and fluid‐attenuated inversion recovery (FLAIR) sequences, and contrast‐enhanced angiography of the brain also revealed no abnormalities, especially no signs of encephalitis. EEG showed a marked slowing of the background rhythm, suggestive of encephalopathy, and spike waves corresponding with seizure activity (Figure 1).

Based on new‐onset seizures, an EEG suggestive of encephalopathy, elevated anti‐TPO and anti‐TG antibody levels, and otherwise extensive and negative workup, a diagnosis of HE was considered.

The patient was started methylprednisolone, 500 mg for 5 days, which was then gradually switched to oral prednisone, 60 mg/day during which time his psychiatric symptoms such as hallucinations and agitation were resolved by haloperidol and olanzapine, respectively. Diazepam, midazolam, sodium valproate, chloral hydrate, and phenobarbital were ineffective at controlling his seizures. His seizures were finally controlled with a combination of sodium valproate 2,400 mg/day, clonazepam 4 mg/day, topiramate 350 mg/day, and levetiracetam 1,800 mg/day.

At his 18‐month follow‐up, the patient has remained seizure‐free and in good physical condition.

## 3 DISCUSSION

Hashimoto's encephalopathy is a rare disease, and the pathophysiology is not known. It can affect people of all ages, but the mean age of onset is about 40 years old, and approximately 75% of cases are in females.^1^ Past reports suggest that the mean age of presentation in children ranges from 12 to 14 years, and the mean age in adults ranges from 45 to 55 years.^7^, ^8^

The clinical presentation of HE is variable; however, the hallmark is a nonspecific encephalopathy characterized by alteration of mental status and consciousness ranging from confusion and/or impaired cognitive function to coma.^1^, ^7^ Based on case series in the literature, presentations in adults include cognitive impairment (36% to 100% of cases), altered consciousness (36% to 85%), transient aphasia (73% to 80%), sleep abnormalities (55%), headache (13% to 50%), neurological deficits (18% to 31%), gait ataxia (28% to 65%), tremors (28% to 84%), myoclonus (37% to 65%), seizures (52% to 66%), and status epilepticus (12%).^4^, ^5^, ^7^, ^9^, ^10^, ^11^, ^12^, ^13^, ^14^ Acute psychosis is the most common psychiatric presentation of HE (26%), followed by depressive disorders (245).^15^ Approximately 80% of children with HE present with new‐onset seizures without other neurological abnormalities C. Overall, approximately 12% of patients with HE present with new‐onset status epilepticus.^1^

Kothbauer‐Magreiter et al^3^ described 2 distinct presentations of HE and thus proposed 2 subtypes. One subtype, vasculitic HE, is characterized by repetitive stroke‐like episodes manifesting as aphasia, hemiparesis, and ataxia with mild cognitive impairment. The other subtype, diffuse HE, is characterized by an insidious and progressive deterioration of mental functions. Seizures can occur in both types.^1^ A meta‐analysis study by de Holanda et al ^4^ including 130 HE patients from 52 reports found that 77 patients (59%) experienced seizures (63 had generalized seizures and 14 had partial seizures). Of the 130 patients, 60% with normal thyroid function, 62% with subclinical hypothyroidism, 80% with overt hypothyroidism, and 56% of patients with hyperthyroidism had seizures.

The importance of diagnosing HE in patients presenting with status epilepticus is essential considering that the etiology of status epilepticus is unknown in 23% of cases, and the short‐term mortality rate of status epilepticus 7% to 22%.^6^ Status epilepticus mostly occurs in adults with HE, while it is not common in children with HE.^1^

The diagnosis of HE is one of exclusion.^1^ Brain MRI and CT, EEG, lumbar puncture and examination of cerebrospinal fluid (CSF), and blood laboratory testing are all necessary to exclude other causes of encephalopathy and seizures such as space‐occupying lesions, Creutzfeldt‐Jakob disease, stroke, vasculitis, metabolic disorders, poisoning, and mental/psychiatric disorders.^1^ The presence of anti‐thyroid antibodies is diagnostic of HE; however, their role in the pathophysiology of the disease is not known.^1^

The first‐line treatments for HE include high‐dose glucocorticoids, intravenous immunoglobulin, and plasma exchange.^1^ When a response to first‐line treatments is poor, second‐line treatments include rituximab and cyclophosphamide.^1^ In most patients, common anti‐seizure medications are ineffective.^1^ Our patient responded well to steroids for control of his symptoms, but common medications used to control seizures were ineffective. His seizures were ultimately controlled by a combination of sodium valproate, clonazepam, topiramate, and levetiracetam. Visée et al^2^ reported that phenytoin, phenobarbital, levetiracetam, lacosamide, and midazolam were ineffective at controlling seizures in a patient with HE. Hilberath et al^7^ reported HE in an adolescent in whom a combination of midazolam, etomidate, thiopental, and diazepam was required to control generalized tonic‐clonic seizure activity. McGinley et al^16^ reported the case of a 42‐year‐old woman with HE who presented in status epilepticus: An anesthetic dose of sodium thiopentone (20 mg/kg) and intravenous dexamethasone (8 mg every 8 hours) was required to control the seizures. The seizures in our patient may be easier to control if he had been on a higher dosage of steroid therapy and for a longer period.^1^, ^17^

Refractory status epilepticus (RSE) refers to continued clinical or electrographic seizures after an adequate initial benzodiazepine dose, followed by a second‐line antiepileptic drug (AED). Non‐drug treatments can be considered for RSE, such as surgery, and neuromodulation treatment. The purpose of epilepsy surgery is to complete resection of an epileptogenic lesion to control seizures.^18^ However, the imaging examinations showed no obvious epileptogenic lesions in our patient, and a non‐lesion patient may not achieve sustained seizure freedom after surgery.^19^ Nevertheless, other non‐drug treatment, such as vagus nerve stimulation (a neuromodulation method)^20^ and next‐generation sequencing,^21^ could also be considered for the treatment of seizures in this patient. Importantly, RSE is associated with a worse prognosis and higher morbidity and mortality than non‐RSE.^6^ Our patient was considered to have RSE. When RSE occurs in an individual without a history of epilepsy and no immediate underlying etiology is identified, it is referred to as new‐onset RSE.^6^ New‐onset RSE is difficult to treat, and the most commonly identified etiologies of new‐onset RSE are autoimmune conditions (19%) and paraneoplastic encephalitis (18%).^6^ HE should be included in the differential diagnosis of RSE.

## 4 CONCLUSIONS

Hashimoto's encephalopathy is a rare disease that should be considered in patients with new‐onset seizures and RSE, and mental status changes when standard investigations are negative. Patients typically respond well to intravenous steroids and anti‐seizure drugs.

## CONFLICT OF INTEREST

All authors disclose that they have no conflicts of interest.

## Data Availability

All the data and materials have been presented in the main paper.
